# HY Zeolite-Supported Monometallic Oxide Catalysts and Insight into the Mechanism of Chlorobenzene Decomposition via Thermal Catalysis

**DOI:** 10.3390/nano16090531

**Published:** 2026-04-28

**Authors:** Peng Sun, Ziwang Zhao, Shuai Ran, Chunyu Wang, Yimeng Liu, Ziyan Wang, Piaoping Yang, Shuyuan Zhou, Yanchun Dong

**Affiliations:** 1 Key Laboratory of Superlight Materials and Surface Technology, Ministry of Education, College of Materials Science and Chemical Engineering, Harbin Engineering University, Harbin 150001, China; 2 State Key Laboratory of Chemistry for NBC Hazards Protection, Beijing 102205, China

**Keywords:** catalytic oxidation, chlorobenzene, HY zeolite, coking resistance, solid-state ion exchange

## Abstract

Here, we report a highly efficient and stable catalytic system based on monometallic oxides supported on HY zeolites for the catalytic oxidation of chlorobenzene (CB). Among the transition and rare-earth metal oxides screened, the 30Cu/HY catalyst demonstrates exceptional performance, achieving near 100% CB conversion at 300 °C (500 ppm CB, 10,000 h^−1^) alongside outstanding 24 h continuous stability without deactivation. Quantitative Py-IR analysis reveals that this superior activity is fundamentally driven by extensive solid-state ion exchange, forming robust Lewis acid centers (Cu-Y structures) that synergize with zeolitic Brønsted acid sites to efficiently polarize and cleave C-Cl bonds. Through an integrated approach combining in situ DRIFTS, real-time mass spectrometry, TGA, and NLDFT pore size analysis, we elucidate that the exceptional deep-oxidation capability of Cu/HY continuously mineralizes carbonaceous intermediates. This property minimizes coke deposition (2.91 wt%) and preserves the hierarchical pore architecture, preventing the coverage of active sites and severe pore blockage by partially oxidized intermediates (such as phenolic, aldehydic, and quinonic species) and stable carbonate species responsible for the deactivation of other metal oxides. These insights provide a mechanistic framework for the rational design of robust, chlorine-resistant catalysts for the sustainable abatement of persistent organic pollutants.

## 1. Introduction

Chlorinated volatile organic compounds (Cl-VOCs), prevalent in construction and decoration materials as volatile gases at ambient temperature, pose substantial risks to human health and the environment due to their persistence, bioaccumulative nature, and toxicity [1,2,3]. The critical challenge in their elimination is the development of catalysts that combine high activity, selectivity, and resistance to chlorine poisoning. The mineralization of carbon to CO_2_ via reactive oxygen species offers an effective pathway for complete pollutant abatement [4]. Thermal catalytic decomposition, which utilizes heated solid catalysts to lower the activation energy for oxidation, operates effectively at moderate temperatures (200–400 °C). This represents a significant energy saving of 300–500 °C compared to direct combustion [5]. A key challenge, however, is incomplete oxidation at lower temperatures, which can lead to the formation of secondary pollutants often more toxic than the parent compounds. Recent studies categorize these by-products into six groups: chlorinated aromatics, chlorinated alkanes/alkenes, oxygenated chlorinated aliphatics, non-chlorinated oxygenated small molecules, oxygenated chlorinated aromatics, and carbon-chlorine radicals [6,7,8]. Notably, compounds such as vinyl chloride (C_2_H_3_Cl), dioxins (2,3,7,8-TCDD) [6], chlorobenzoquinones [7], and epichlorohydrin [8] are of particular concern due to their high toxicity. Maintaining the reaction temperature within a 250–350 °C window is crucial to minimize the generation of these hazardous substances [9,10]. Thus, designing catalysts that achieve high efficiency and selectivity within this specific temperature range, thereby preventing secondary pollution, remains a paramount objective.

Transition metal oxides (TMOs) and rare-earth metal oxides are widely reported as effective catalysts for Cl-VOCs abatement. Typical TMOs, including MnO_x_ [11], Fe_2_O_3_ [12], CuO [13], and Co_3_O_4_ [14], facilitate oxidation through efficient oxygen cycling. Rare-earth oxides like CeO_2_ offer high activity driven by abundant oxygen vacancies, which confer excellent oxygen storage capacity and redox properties [15]. A major limitation of bulk metal oxides, however, is their susceptibility to sintering and deactivation. The latter often results from the formation of stable metal-chlorine (M-Cl) bonds that permanently occupy active sites [16]. To address these limitations and enhance catalyst durability, supporting metal oxides on zeolites has emerged as a superior strategy. Compared to conventional amorphous supports like silica or alumina, zeolites possess a unique crystalline porous framework that offers distinct structural and chemical advantages. First, the remarkable spatial confinement effect of zeolitic channels effectively anchors active metal species, promoting the formation of ultrasmall, highly dispersed nanoparticles while suppressing their severe sintering and unfavorable element migration under harsh thermal conditions [17,18]. Second, zeolites feature highly tunable Brønsted and Lewis acidity. The intimate proximity and strong interaction between the variable-valence metal oxides and the zeolitic acid sites create a powerful bifunctional synergistic effect. This synergy is crucial for simultaneously enhancing target molecule activation, optimizing shape-selectivity, and boosting the overall resistance to halogen poisoning [19]. Building upon these unique traits, zeolites such as H-ZSM-5 [11], Beta [20], and MFI [21] have become favored supports, wherein their inherent acidity synergizes with the redox properties of the active phase to maximize catalytic performance. For instance, a study on H-ZSM-5, H-BEA, and H-MOR for trichloroethylene (TCE) oxidation revealed that deactivation correlated with the loss of hydroxyl groups linked to strong Brønsted acid sites, lowering HCl and CO_2_ selectivity. This could be mitigated by introducing H_2_O, which regenerates protonic sites and facilitates chlorine removal as HCl [22]. For example, Lin et al. reported that MnO_x_ supported on H-ZSM-5 exhibited superior activity for oxidizing CB, 1,2-dichloroethane (DCE), and TCE, attributing this to the zeolite’s synergistic combination of acid sites and microporous structure, which enhances adsorption and mineralization [11]. Conversely, optimizing the active phase loading is critical. Liu et al. found that while Cu/H-ZSM-5 is active for DCE oxidation, high Cu loadings promote the formation of bulky CuO_x_ clusters leading to rapid initial deactivation, whereas low loadings result in deactivation via coking [13]. Similarly, Shi et al. achieved high DCE oxidation activity (T_90_ = 255 °C) with a Cr-Pt/ZSM-5 catalyst but noted that excessive Cr loading degraded the zeolite framework and caused metal aggregation [23]. Sun et al. demonstrated that a bimetallic Cu-Nb/H-ZSM-5 catalyst prepared by impregnation was effective for CB oxidation and could be regenerated after deactivation at 400 °C [24]. These studies underscore the importance of the synergistic interplay between the redox-active metal centers and the zeolite’s acidic properties in designing robust catalysts for Cl-VOC removal.

In this study, we prepare a series of catalysts comprising variable-valence metal oxides (CuO, CeO_2_, MnO_x_, Fe_2_O_3_) supported on HY zeolite via impregnation, with metal loadings ranging from 5–40 wt%. Their catalytic performance for CB oxidation is systematically evaluated in terms of activity, stability, and CO_2_ selectivity. A suite of characterization techniques, including XRD, SEM, XPS, H_2_-TPR, and O_2_-TPD, is employed to elucidate their physicochemical properties. Furthermore, the reaction mechanism, intermediate pathways, and causes of deactivation are investigated through mass spectrometry and in situ DRIFTS.

## 2. Materials and Methods

### 2.1. Catalyst Preparation

The supported catalysts were prepared via a wet impregnation method. Precursor solutions were obtained by dissolving calculated amounts of Cu(NO_3_)_2_, Ce(NO_3_)_3_·6H_2_O, Mn(NO_3_)_2_·4H_2_O, and Fe(NO_3_)_3_·9H_2_O (Shanghai Macklin Biochemical Co., Ltd., Shanghai, China) in 20 mL of anhydrous ethanol. Then, 2 g of HY zeolite (SiO_2_/Al_2_O_3_ molar ratio = 12, Zhuoran Environmental Protection Co., Ltd., Shenzhen, China) was added to the solution. The nominal metal loadings were defined as the mass percentage of the metal element relative to the mass of the HY support (i.e., 5, 10, 20, 30, and 40 wt%). Consequently, the corresponding mass of metal atoms used was 0.1, 0.2, 0.4, 0.6, and 0.8 g, respectively. The resulting slurries were stirred at room temperature for 6 h to ensure adsorption equilibrium. Subsequently, the resulting residues were dried in an oven at 100 °C for 12 h. The dried powders were calcined in static air at 550 °C for 4 h (heating rate: 10 °C/min). Finally, the catalysts were ground and denoted as xM/HY (x = 5–40; M = Cu, Ce, Mn, Fe). The HY support was treated under identical conditions for reference. Prior to evaluation, all samples were pelletized, crushed, and sieved to a 20–40 mesh size.

### 2.2. Catalytic Activity Evaluation

Catalytic performance for CB decomposition was evaluated in a laboratory-scale fixed-bed reactor. A 500 ppm CB gas stream was generated by passing one flow of compressed air through a temperature-controlled bubbler containing liquid CB. This stream was subsequently diluted and homogenized with a second air flow in a mixing chamber. The concentration was precisely regulated by adjusting both the water bath temperature of the bubbler and the flow rates of the two air streams. The total gas flow corresponded to a GHSV of 10,000 h^−1^. For each test, the catalyst (20–40 mesh) was loaded into a quartz tubular reactor, yielding a fixed catalyst bed height of 4 cm. The reactor temperature was programmable from 30 to 450 °C. All gas lines and the mixing chamber were heated to 100 °C to prevent any condensation of CB. The composition of the effluent gas was monitored online using a gas chromatograph (SHIMADZU GC-2014C, Kyoto, Japan) equipped with both a flame ionization detector (FID) and a thermal conductivity detector (TCD). A DB-FFAP capillary column was installed. CB concentration was quantified using the FID, while CO_2_ was analyzed using the TCD. Blank experiments (i.e., without catalyst) confirmed the thermal stability of CB under the applied reaction temperatures, ensuring that any conversion was attributable to catalytic action. CB conversion and CO_2_ selectivity were calculated using the following Equations (1) and (2), respectively, where *C_CB-in_* and *C_CB-out_* are the inlet and outlet concentrations of CB, and *C_CO2-out_* and *C_CO2-Air_* are the outlet CO_2_ concentration and the background CO_2_ concentration in the feed air, respectively. The factor 6 corresponds to the stoichiometric number of CO_2_ molecules produced from the complete oxidation of one CB molecule.

CB conversion = (1 − *C_CB-out_/C_CB-in_*) × 100% (1)

CO_2_ selectivity = (*C_CO2-out_* − *C_CO2-Air_*)/[(*C_CB-in_* − *C_CB-out_*) × 6] × 100% (2)

### 2.3. Catalyst Characterization

Detailed experimental protocol (XRD, SEM, TEM, XPS, etc. analysis) used for the characterization of developed catalysts is provided in the Supplementary Material.

### 2.4. In Situ DRIFTS Experiments

In situ *DRIFTS* experiments were performed on a Bruker INVENIO S spectrometer. Spectra were recorded in the range of 800–4000 cm^−1^ with a resolution of 8 cm^−1^, accumulating 64 scans per spectrum. Prior to reaction, the catalyst sample was loaded into a high-temperature DRIFTS cell and pretreated under a 50 mL/min Ar flow while heating to 350 °C at 10 °C/min. Background spectra were collected at selected temperatures between 200 and 350 °C. After cooling to room temperature, the reactant gas mixture (500 ppm CB and 20% O_2_, balanced with Ar) was introduced at a total flow rate of 50 mL/min. The temperature was then ramped according to the desired program, and spectra were acquired at specific temperatures to monitor the evolution of surface species.

## 3. Results and Discussion

### 3.1. Selection of the Active Metal Oxide Component

**Figure 1:**
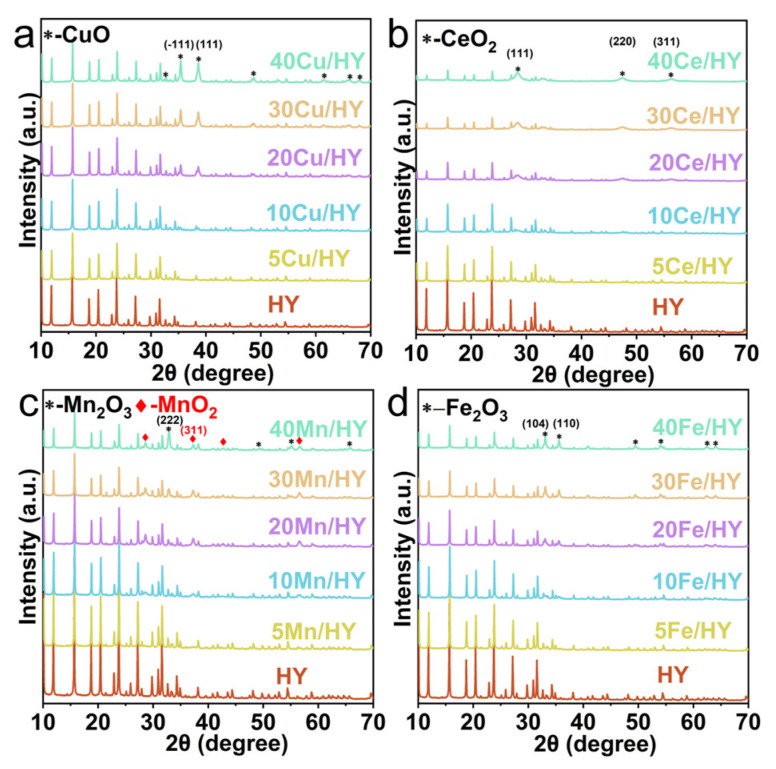
XRD patterns of HY and xM/HY catalysts (x = 5, 10, 20, 30, 40; M = Cu (**a**), Ce (**b**), Mn (**c**), Fe (**d**)).

Figure 1 presents the X-ray diffraction (XRD) patterns of the synthesized xM/HY catalysts (x = 5, 10, 20, 30, 40; M = Cu, Ce, Mn, Fe). All observed diffraction peaks can be indexed to the crystalline phases of the HY zeolite support (JCPDS 45-0112) and the respective metal oxides—CuO (JCPDS 80-1916), CeO_2_ (JCPDS 34-0394), Mn_2_O_3_ (JCPDS 24-0508), MnO_2_ (JCPDS 42-1169), and Fe_2_O_3_ (JCPDS 33-0644). The absence of extraneous peaks confirms the successful preparation of the intended series of supported metal oxide catalysts.

**Figure 2:**
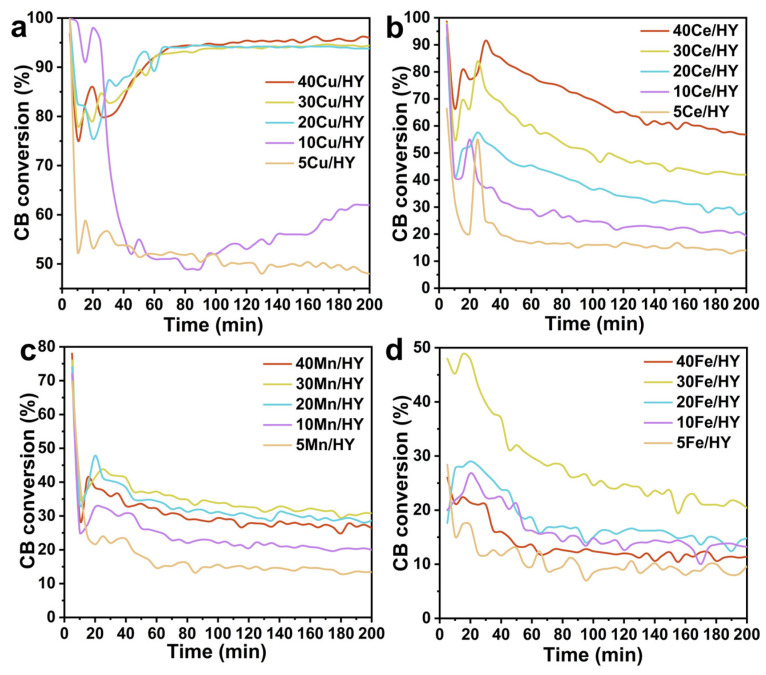
Catalytic performance of xM/HY catalysts (x = 5, 10, 20, 30, 40; M = Cu (**a**), Ce (**b**), Mn (**c**), Fe (**d**)) within 200 min at 300 °C. Conditions: 500 ppm CB, GHSV = 10,000 h^−1^.

To identify the most effective active component for CB decomposition, we evaluated the catalytic performance of a series of xM/HY catalysts (x = 5, 10, 20, 30, 40; M = Cu, Ce, Mn, Fe). Figure 2 presents the CB conversion as a function of time on stream (up to 200 min) for these catalysts. For the Cu/HY series (Figure 2a), all catalysts exhibited an initial conversion ≥99%, demonstrating that the initial activity is independent of the Cu loading. However, a rapid deactivation was observed for the catalysts with lower loadings: the conversion for 5Cu/HY and 10Cu/HY dropped steeply to ~40% within the first 30 min for CB, respectively. In stark contrast, catalysts with higher Cu loadings (20–40 wt%) maintained stable and high activity, sustaining ~97% conversion. This indicates that a lower CuO loading results in a sparser distribution of active sites, rendering the catalyst more susceptible to rapid deactivation. For the Ce/HY series (Figure 2b), the catalytic activity increased progressively with higher metal loadings in the 20–40 wt% range. In the cases of Mn/HY and Fe/HY, the optimal loading was found to be 30 wt%; however, even at this optimum, their CB conversion levels remained lower than those achieved by 30Cu/HY and 30Ce/HY catalysts. This comparative analysis identifies Cu/HY and Ce/HY as the most active systems among the four metal oxides screened. Furthermore, an examination of the entire dataset in Figure 2 highlights the exceptional catalytic stability of the Cu/HY catalysts throughout the 200 min test. Based on these activity and stability assessments, Cu/HY and Ce/HY were selected as the most promising catalyst systems. Therefore, the catalysts of 30M/HY (M = Cu, Ce, Mn, Fe) were selected for subsequent comparative characterization and mechanistic studies.

### 3.2. Analysis of Catalyst Morphology

**Figure 3:**
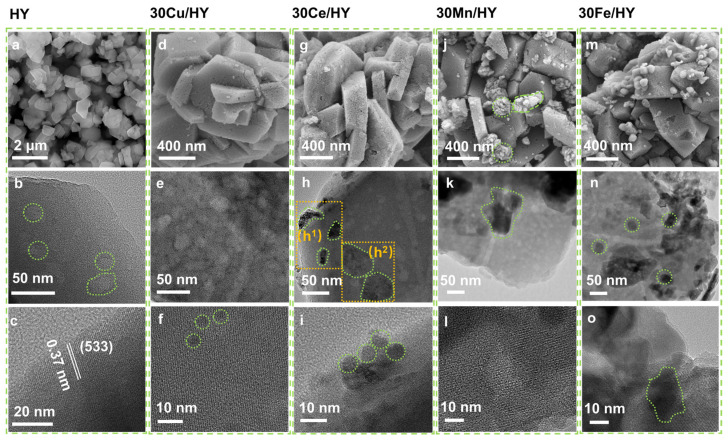
SEM and TEM images of HY (**a**–**c**), 30Cu/HY (**d**–**f**), 30Ce/HY (**g**–**i**), 30Mn/HY (**j**–**l**), and 30Fe/HY (**m**–**o**).

Figure 3 presents representative scanning and transmission electron microscopy (SEM/TEM) images of the HY zeolite and the 30M/HY catalysts (M = Cu, Ce, Mn, Fe). The HY support (Figure 3a) displays an irregular morphology consisting of flakes and particles with dimensions between 200 nm and 1 μm, providing ample sites for metal anchoring. TEM analysis at lower magnification (Figure 3b) reveals a highly porous architecture with irregular pore sizes predominantly in the 10–50 nm range. This extensive porosity affords a high specific surface area, promoting efficient diffusion of reactant molecules to internal active and acid sites [25]. High-resolution TEM (HRTEM, Figure 3c) confirms the crystalline nature of HY, showing lattice fringes with a measured d-spacing of 0.37 nm, indexed to the (533) plane. The SEM image of 30Cu/HY (Figure 3d) shows that CuO nanoparticles are deposited on the zeolite flakes without severe large-scale aggregation. TEM images at different magnifications (Figure 3e,f) reveal that the CuO particles exhibit better dispersion performance on the HY surface with an average size of approximately 5 nm. Due to the small particle size, the lattice fringes of CuO observed in high-resolution transmission electron microscopy (HRTEM) images are not distinct; however, this nanometric dimension is conducive to exposing a greater number of active sites, thereby enhancing catalytic activity. For 30Ce/HY, the SEM image (Figure 3g) indicates a tendency for CeO_2_ to form agglomerates on the zeolite surface. This is corroborated by higher-magnification TEM (Figure 3h,i), which reveals a non-uniform distribution; some regions (e.g., h^1^) show significant aggregation, while others (e.g., h^2^) retain a relatively good dispersion of CeO_2_ particles. This suggests a coexistence of agglomerated and well-dispersed CeO_2_ phases on the support. In stark contrast, both MnO_x_ and Fe_2_O_3_ displayed pronounced aggregation and non-uniform distribution on the HY support (Figure 3j,m). TEM analysis at varying magnifications (Figure 3k,l for 30Mn/HY; Figure 3n,o for 30Fe/HY) confirms the formation of large, irregular clusters of metal oxides with no defined morphology. This severe aggregation likely contributes to their inferior catalytic performance. We speculate that during impregnation, Mn^2+^ and Fe^3+^ ions may compete less effectively with the framework protons (H^+^) of HY for exchange sites compared to Cu^2+^ or Ce^3+^, leading to preferential deposition and growth on the external surface rather than within the zeolite channels [26]. Elemental mapping by energy-dispersive X-ray spectroscopy (EDS, Figure S1) provides further evidence: Cu and Ce elements are uniformly distributed across the HY support, whereas Mn and Fe signals are concentrated in dense clusters. The improved dispersion and uniform distribution of CuO and CeO_2_ facilitate the optimal exposure of active sites, serving as a critical factor for their superior catalytic performance.

### 3.3. Specific Surface Area and Elemental Content of Catalyst

The textural properties of the catalysts were investigated by N_2_ physisorption. The Brunauer–Emmett–Teller (BET) surface areas (S_BET_), derived from the isotherms shown in Figure S2, are compiled in Table 1. Loading with metal oxides significantly reduces the S_BET_ from 686.47 m^2^/g for HY to 416.06 (30Cu/HY), 408.24 (30Ce/HY), 387.26 (30Mn/HY), and 385.69 m^2^/g (30Fe/HY). This decrease is expected and can be primarily ascribed to two factors: (i) the partial occupation or blocking of the micropores by the deposited metal oxide nanoparticles, and (ii) potential structural alterations or partial collapse of the zeolite framework due to interaction with the metal precursors during calcination [27]. Notably, the more pronounced S_BET_ reduction for Mn/HY and Fe/HY aligns with their observed severe metal oxide aggregation on the external surface (Figure 3j,m), which contributes less to the internal porosity. Furthermore, it is critical to highlight the distinct but synergistic roles of microporosity and mesoporosity in the catalytic oxidation of CB. The native micropores (<2 nm) of the HY zeolite provide a robust spatial confinement effect and host a high density of Brønsted acid sites, which serve as the primary micro-environments for the strong adsorption and initial electrophilic activation of the CB molecules. However, relying solely on micropores often leads to severe mass-transfer limitations for the bulky aromatic intermediates generated during the reaction. The presence of mesopores (2–50 nm), primarily arising from the inter-particle voids formed during metal oxide loading, addresses this limitation by providing efficient diffusion pathways. These mesoporous channels significantly enhance the internal diffusion kinetics, facilitating the rapid migration of bulky intermediates to accessible active sites for complete mineralization, or allowing their timely desorption before they polymerize into polyaromatic coke. Therefore, maintaining this hierarchical (micro-mesoporous) pore architecture is a prerequisite for achieving sustained catalytic activity and preventing rapid deactivation.

The actual metal loadings of the 30M/HY (M = Cu, Ce, Mn, Fe) catalysts were determined by inductively coupled plasma optical emission spectrometry (ICP-OES). Table 1 compares the measured values (*C_a_*) with the theoretical metal content (*C_t_*), the latter calculated assuming complete conversion of the precursors to their respective oxides (CuO, CeO_2_, Mn_2_O_3_, Fe_2_O_3_). The measured loadings (19.18% Cu, 21.35% Ce, 18.03% Mn, 20.93% Fe) are consistently lower than the corresponding theoretical values (21.81% Cu, 21.92% Ce, 20.97% Mn, 20.99% Fe). This minor deviation is commonly observed for impregnated zeolite catalysts and can be attributed to losses during drying and calcination, such as precursor migration or incomplete retention on the support.

### 3.4. Redox Properties of the Catalyst

**Figure 4:**
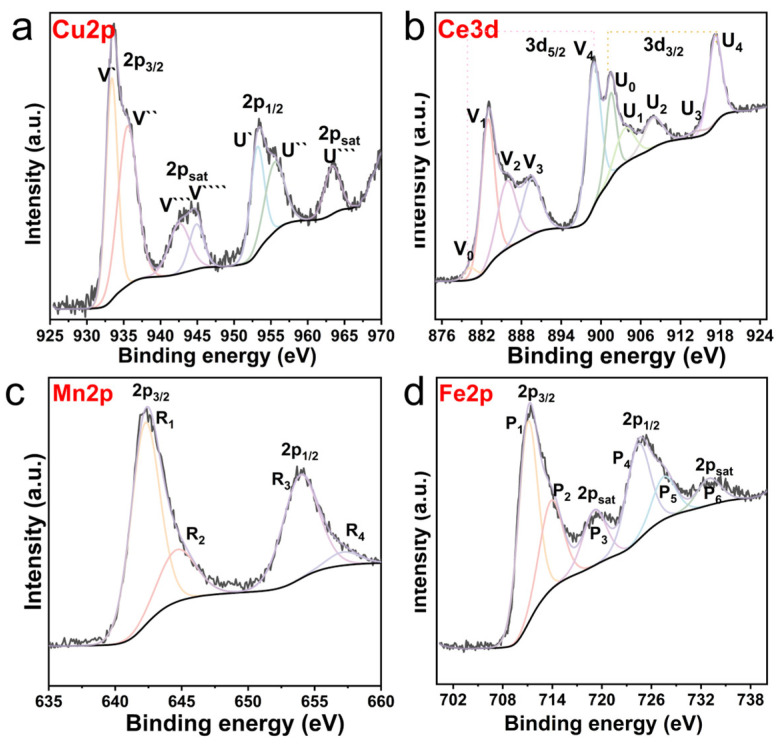
XPS spectra of 30M/HY catalysts (M = Cu, Ce, Mn, Fe); Cu 2p spectra (**a**), Ce 3d spectra (**b**), Mn 2p spectra (**c**), and Fe 2p spectra (**d**).

The surface chemical states of the active metals in the 30M/HY (M = Cu, Ce, Mn, Fe) catalysts were investigated by X-ray photoelectron spectroscopy (XPS), with deconvoluted spectra and peak assignments presented in Figure 4. The Cu 2p spectrum for the 30Cu/HY catalyst (Figure 4a) was deconvoluted into multiple components. The main spin–orbit doublet (Cu 2p_3/2_ at 933.58 eV and Cu 2p_1/2_ at 953.38 eV) along with its pronounced shake-up satellite features are characteristic of Cu^2+^ species. A minor component at a slightly lower binding energy (~933.4 eV) suggested the presence of reduced copper (Cu^+^/Cu^0^). Since Cu 2p spectra alone cannot unambiguously discriminate between Cu^+^ and Cu^0^, complementary Cu LMM Auger electron spectroscopy was performed (Figure S3). The Auger parameter confirmed the existence of Cu^+^ on the catalyst surface. The Ce 3d spectrum for 30Ce/HY (Figure 4b) exhibits the complex multiplet structure typical of cerium oxides. Deconvolution yields ten peaks, conventionally labeled V and U for the 3d_5/2_ and 3d_3/2_ spin–orbit doublets, respectively. Peaks labeled V_0_, V_3_, V_4_, U_0_, U_3_, and U_4_ are characteristic of Ce^4+^ species (3d^10^4f^0^ final state), while peaks V_1_, V_2_, U_1_, and U_2_ are attributed to Ce^3+^ species (3d^10^4f^1^ final state), indicating a mixed Ce^4+^/Ce^3+^ valence state. The Mn 2p spectrum for 30Mn/HY (Figure 4c) was fitted with four components. The Mn 2p_3/2_ and Mn 2p_1/2_ envelopes suggest the presence of mixed manganese oxidation states, primarily Mn^3+^ and Mn^4+^. This finding corroborates the XRD analysis (Figure 1), which identified the co-existence of Mn_2_O_3_ and MnO_2_ crystalline phases. The Fe 2p spectrum for 30Fe/HY (Figure 4d) was deconvoluted into six peaks within the Fe 2p_3/2_ and Fe 2p_1/2_ regions. The peak positions and their relative intensities indicate the co-existence of both Fe^2+^ and Fe^3+^ oxidation states on the catalyst surface, consistent with the reported values for iron oxides [28,29]. In summary, XPS analysis reveals that the surface metal species in all four M/HY catalysts exist in mixed oxidation states (Cu^2+^/Cu^+^, Ce^4+^/Ce^3+^, Mn^4+^/Mn^3+^, Fe^3+^/Fe^2+^). The presence of lower-valent cations is intrinsically linked to the formation of oxygen vacancies within the metal oxide phases. These vacancies are crucial for enhancing lattice oxygen mobility and facilitating the redox cycles that underpin the catalytic activity in oxidation reactions, highlighting a key merit of using metal oxides as active components.

**Figure 5:**
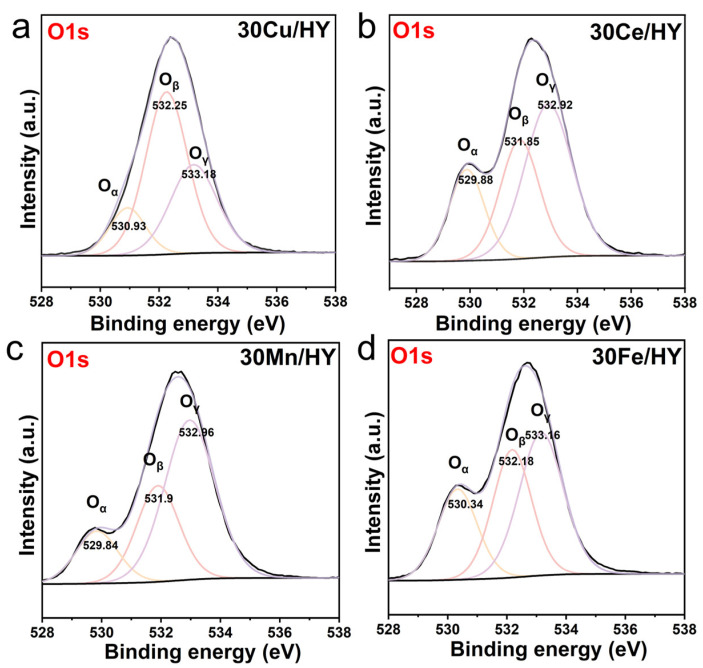
O1s XPS spectra of 30M/HY catalysts (M = Cu (**a**), Ce (**b**), Mn (**c**), Fe (**d**)).

The chemical nature of oxygen species on the catalyst surfaces was further investigated by XPS. As shown in Figure 5, the O1s spectra for all four 30M/HY catalysts (M = Cu, Ce, Mn, Fe) were deconvoluted into three distinct components, denoted as O_α_, O_β_, and O_γ_. The O_α_ peak is attributed to lattice oxygen within the metal oxides. The O_β_ component corresponds to surface-adsorbed oxygen species, including reactive oxygen (e.g., O^−^, O_2_^−^), oxygen vacancies, and surface hydroxyl groups (-OH). The O_γ_ peak is assigned to physically adsorbed water and the framework oxygen (Si-O-Si, Si-O-Al) of the HY zeolite. Notably, the surface-active oxygen species represented by the O_β_ component (such as oxygen vacancies and chemisorbed oxygen) are strictly crucial for catalytic oxidation, as they act as the primary active sites for the deep mineralization of reactant molecules. To quantitatively compare the abundance of these crucial species, the relative atomic ratios of the oxygen components were calculated and summarized in Table 1. Remarkably, the 30Cu/HY catalyst exhibits the highest proportion of surface-active oxygen (O_β_/O_all_ = 0.54), significantly surpassing those of 30Fe/HY (0.33), 30Ce/HY (0.31), and 30Mn/HY (0.28). This exceptionally high relative concentration of reactive oxygen species on the outermost layer of 30Cu/HY ensures a continuous and abundant supply of highly reactive surface oxygen.

**Figure 6:**
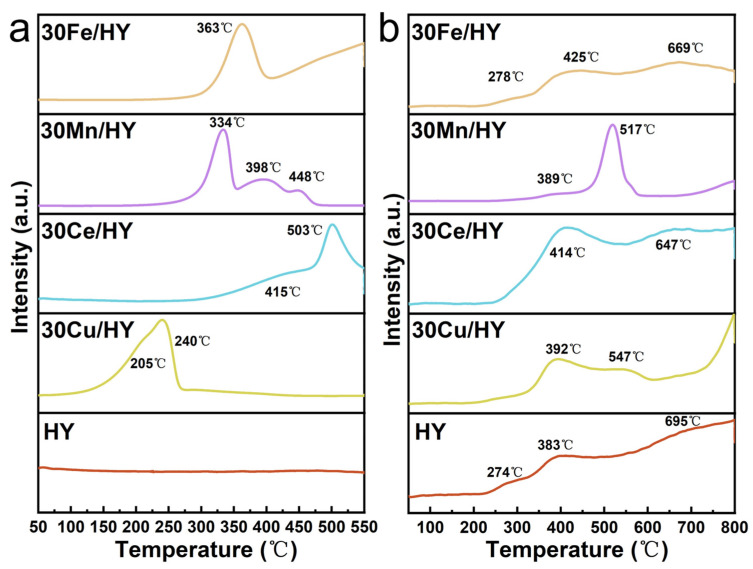
(**a**) H_2_-TPR and (**b**) O_2_-TPD curves of HY and 30M/HY catalysts (M = Cu, Ce, Mn, Fe).

The redox properties and oxygen mobility of the catalysts were probed by H_2_ temperature-programmed reduction (H_2_-TPR) and O_2_ temperature-programmed desorption (O_2_-TPD) experiments, as shown in Figure 6a and Figure 6b, respectively. The H_2_-TPR profile of the HY zeolite (Figure 6a) shows no reduction features up to 550 °C, confirming its redox inactivity. In contrast, distinct reduction peaks appear for all metal oxide-loaded catalysts. For 30Cu/HY, a broad, asymmetric reduction feature is observed between 100 and 250 °C, with a maximum at 240 °C and a distinct shoulder at 205 °C. This profile signifies multiple CuO species with different dispersions and interactions with the support. The low-temperature shoulder (205 °C) is assigned to the reduction of highly dispersed CuO clusters strongly interacting with the zeolite framework. The main peak at 240 °C corresponds to the reduction of larger, yet well-dispersed, CuO nanoparticles (Cu^2+^ → Cu^0^) [30]. The H_2_-TPR profile of 30Ce/HY features two peaks at 415 °C and 503 °C, characteristic of ceria reduction. The lower-temperature peak is typically assigned to the removal of surface-adsorbed and labile lattice oxygen (e.g., O_2_^2−^/O^−^), while the peak at 503 °C corresponds to the reduction of bulk-like (subsurface) oxygen and the associated reduction of Ce^4+^ to Ce^3+^ [31]. The reduction of 30Mn/HY proceeds in three consecutive steps, with peaks at 334, 398, and 448 °C. This stepwise profile is consistent with the sequential reduction of mixed manganese oxides (MnO_2_ and Mn_2_O_3_) as follows: MnO_2_ → Mn_2_O_3_ (334 °C), Mn_2_O_3_ → Mn_3_O_4_ (398 °C), and Mn_3_O_4_ → MnO (448 °C) [32]. In contrast, the H_2_-TPR profile of 30Fe/HY presents a single, broad reduction peak centered at 363 °C. This is characteristic of the overlapping, multi-step reduction of Fe_2_O_3_ to metallic Fe (Fe_2_O_3_ → Fe_3_O_4_ → FeO → Fe^0^) [33], consistent with the hematite phase identified by XRD. The broadness of the peak indicates a distribution of particle sizes or reduction kinetics. A comparative analysis of the H_2_-TPR profiles (Figure 6a) reveals that the 30Cu/HY catalyst initiates reduction at the lowest temperature among the monometallic catalysts. This lower reduction temperature signifies a higher intrinsic redox activity, which correlates well with its superior low-temperature catalytic performance.

The O_2_-TPD profile of the HY zeolite (Figure 6b) exhibits three desorption features at 274, 383, and 695 °C. The low-temperature peak (274 °C) is assigned to the desorption of physisorbed or weakly bound molecular oxygen (O_2_). The peak at 383 °C likely corresponds to the removal of hydroxyl groups associated with framework defects. The high-temperature peak at 695 °C is attributed to the evolution of lattice oxygen from the zeolite framework, a process potentially linked to severe dealumination and structural rearrangement at elevated temperatures [34]. For 30Cu/HY, two distinct O_2_ desorption peaks are observed at 392 and 547 °C (Figure 6b). The lower-temperature peak (392 °C) is characteristic of desorbing surface chemisorbed oxygen species, which are crucial as active oxygen sources for low-temperature oxidation. The prominence of this peak aligns with the catalyst’s facile reducibility observed in H_2_-TPR (main peak at ~240 °C), collectively underscoring its high low-temperature redox activity [35]. The higher-temperature peak (547 °C) is associated with the release of strongly bound lattice oxygen (O^2−^) from bulk-like CuO or from interfacial sites between CuO and the zeolite support [36]. The O_2_-TPD profile of 30Ce/HY features two peaks at 414 and 647 °C. The peak at 414 °C is assigned to surface-adsorbed reactive oxygen, which serves as the primary active oxygen pool for catalytic reactions on ceria. The high-temperature peak at 647 °C corresponds to the release of bulk lattice oxygen (O^2−^). Its evolution at such a high temperature indicates a process involving the diffusion of O^2−^ through the lattice via oxygen vacancies and is intimately linked to the reduction of Ce^4+^ to Ce^3+^, reflecting the material’s bulk oxygen mobility and storage capacity [37]. In the case of 30Mn/HY, desorption occurs at 389 and 517 °C. The lower-temperature signal (389 °C) originates from surface chemisorbed oxygen. The dominant peak at 517 °C is attributed to the release of lattice oxygen (O^2−^) from the bulk of the manganese oxide phases (Mn_2_O_3_/MnO_2_) [38], consistent with the mixed oxidation states identified by XPS. The O_2_-TPD profile for 30Fe/HY is complex, showing three peaks at 278, 425, and 669 °C. This multiplicity is characteristic of bulk-like iron oxide (Fe_2_O_3_). The peak at 278 °C is ascribed to weakly bound surface chemisorbed oxygen. The intermediate peak at 425 °C may arise from more strongly bound surface oxygen or the release of oxygen from near-surface layers. The high-temperature peak at 669 °C corresponds to the evolution of bulk lattice oxygen from Fe_2_O_3_, consistent with its reduction profile in H_2_-TPR. A quantitative comparison of the low-temperature O_2_ desorption peaks (representing reactive surface oxygen species) reveals the following order in peak area: 30Ce/HY > 30Cu/HY > 30Fe/HY > 30Mn/HY (Figure 6b). This trend suggests that the CeO_2_ phase in 30Ce/HY possesses the highest capacity for supplying reactive surface oxygen, followed by CuO in 30Cu/HY. However, although 30Ce/HY possesses a larger capacity for reactive oxygen, its higher reduction temperature (observed in H_2_-TPR) limits the utilization of these species at lower temperatures compared to 30Cu/HY. The abundance of these readily available oxygen species correlates directly with the catalytic activity, explaining why 30Ce/HY and 30Cu/HY exhibit superior performance in the low-temperature oxidation of CB compared to their Mn and Fe counterparts.

### 3.5. Brønsted and Lewis Acid Concentrations on the Catalyst

**Figure 7:**
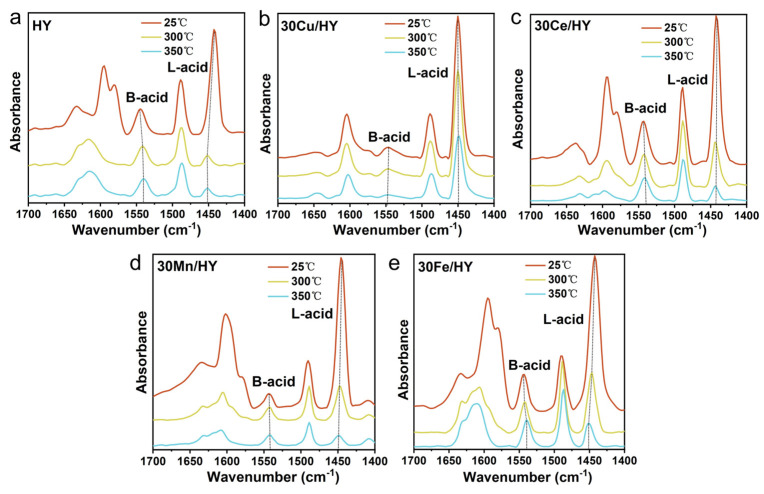
Py-IR spectra of HY and 30M/HY catalysts (M = Cu, Ce, Mn, Fe) recorded at different desorption temperatures (25, 300, and 350 °C): (**a**) HY, (**b**) 30Cu/HY, (**c**) 30Ce/HY, (**d**) 30Mn/HY, and (**e**) 30Fe/HY.

The type, concentration, and thermal stability of acid sites on the catalysts, which are crucial for the adsorption and activation of CB, were evaluated using pyridine-adsorption infrared spectroscopy (Py-IR) at desorption temperatures of 25, 300, and 350 °C. As shown in Figure 7, all spectra exhibit characteristic bands at approximately 1450 cm^−1^ and 1540 cm^−1^, which are assigned to pyridine coordinately bound to Lewis acid (L-acid) sites and pyridinium ions (PyH^+^) adsorbed on Brønsted acid (B-acid) sites, respectively. The quantitative results derived from the peak integrations are summarized in Table 2. The pure HY zeolite is dominated by abundant B-acid sites (199.36 μmol/g at 300 °C) originating from its framework hydroxyls (≡Si–OH–Al≡), with a relatively low L-acid concentration.

Upon the introduction of metal oxides, the acid site distribution undergoes significant alterations. Among the impregnated catalysts, the 30Cu/HY sample presents a striking and highly anomalous acid profile. At the catalytic reaction temperature (300 °C), 30Cu/HY possesses the highest L-acid concentration (143.98 μmol/g) among all samples, accompanied by a drastic depletion of B-acid sites (dropping to merely 18.79 μmol/g) compared to the pure HY support. Consequently, its B/L ratio plummets to 0.13. This anomalous massive consumption of B-acid sites with the concurrent generation of highly stable L-acid sites provides unequivocal evidence for the occurrence of extensive ion exchange during the preparation process [39]. Rather than merely accumulating as bulk CuO particles on the external surface, a substantial fraction of Cu^2+^ precursor ions migrate into the zeolite channels and undergo solid-state ion exchange with the framework protons (H^+^). This interaction replaces the zeolitic Brønsted acid sites with isolated Cu^2+^ or [Cu-OH]^+^ species, thereby forming robust Cu-Y structures [40]. These exchanged copper species act as strong Lewis acid centers that exhibit remarkable thermal stability, retaining a high L-acid concentration (93.35 μmol/g) even at a harsh desorption temperature of 350 °C. In contrast, the 30Mn/HY catalyst exhibits a severe loss of L-acidity at elevated temperatures, dropping to only 25.51 μmol/g at 300 °C. This indicates that the Mn species predominantly exist as bulk manganese oxides (corroborating the XRD and SEM results) which only provide weak surface acidity and fail to construct stable L-acid sites. During the reaction at 300 °C, the abundant and thermally stable L-acid sites (exchanged Cu^2+^) on 30Cu/HY serve as potent anchoring points for the nucleophilic chlorine atom of CB, deeply polarizing the C-Cl bond. Simultaneously, the residual B-acid sites provide the necessary protons for the rapid desorption of dissociated chlorine as HCl. This optimal balance and stability of acid sites distinctly set 30Cu/HY apart as the most efficient catalyst for CB destruction.

### 3.6. Evaluation of Catalytic Stability and Coking Behavior

**Figure 8:**
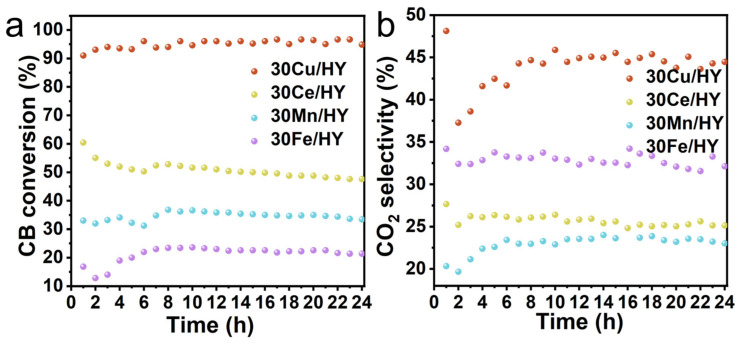
Long-term catalytic performance of 30M/HY catalysts (M = Cu, Ce, Mn, Fe) over 24 h at 300 °C: (**a**) CB conversion and (**b**) CO_2_ selectivity. Conditions: 500 ppm CB, GHSV = 10,000 h^−1^.

To assess the practical application potential of the catalysts, long-term stability tests for CB decomposition were conducted continuously for 24 h at 300 °C. As depicted in Figure 8a, the 30Cu/HY catalyst exhibited exceptional durability. Its CB conversion rapidly reached over 95%, maintaining this remarkable activity without any sign of degradation throughout the entire 24 h period. In parallel, its CO_2_ selectivity steadily increased and stabilized at ~45% (Figure 8b), indicating a sustained and robust deep oxidation capability. In contrast, the 30Ce/HY catalyst suffered from noticeable deactivation, with its conversion dropping from an initial ~60% to ~45%. The 30Mn/HY and 30Fe/HY catalysts exhibited lower steady-state CB conversions of approximately 35% and 24%, respectively, accompanied by inferior CO_2_ selectivities (<35%). To contextualize this exceptional durability, the performance of 30Cu/HY was benchmarked against recently reported state-of-the-art catalysts. For instance, while noble metal-based catalysts (e.g., Ru systems) can achieve CB conversion at lower temperatures, they are notoriously susceptible to rapid chlorine poisoning and structural deactivation within a few hours [41]. Conversely, typical non-noble transition metal oxides (such as bulk Mn/Ce-based systems) generally require significantly higher operating temperatures (often >350 °C) to reach comparable conversions and frequently suffer from severe carbonaceous deposition (coking) during prolonged continuous operation [42,43]. By achieving near 100% CB conversion at a relatively mild 300 °C while maintaining peak performance over a 24 h period, the 30Cu/HY system successfully bridges these critical performance gaps. Although the 30Cu/HY catalyst exhibits superior low-temperature activity for CB conversion, its CO_2_ selectivity (~44%) indicates a need for enhanced deep oxidation capabilities. Future strategies to improve complete mineralization could involve constructing multimetallic synergistic systems. For instance, integrating Cu with Ce and Mn could combine the powerful low-temperature reducibility of Cu, the exceptional oxygen storage capacity of Ce, and the multivalent redox properties of Mn to facilitate deep oxidation. Additional strategies, such as noble metal doping or hierarchical pore engineering of the zeolite support, could also be explored to promote the rapid mass transfer and deep mineralization of bulky intermediate species.

**Figure 9:**
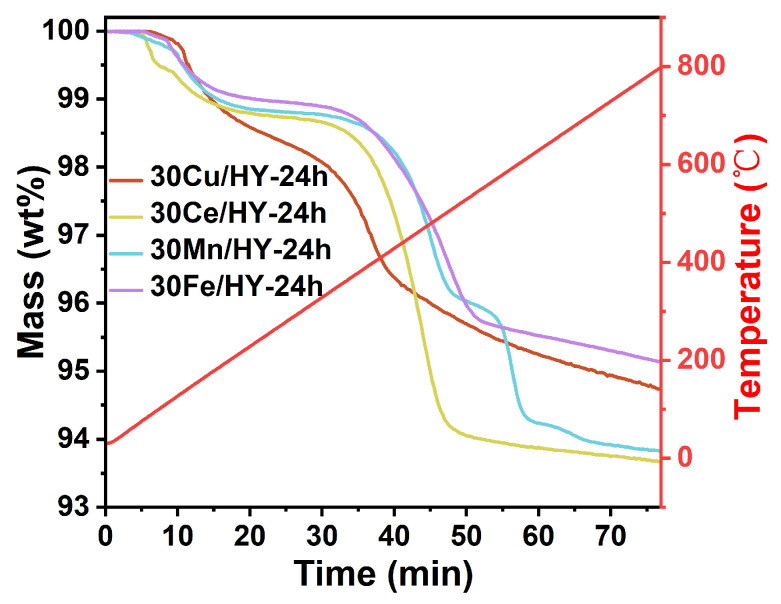
TGA curves of the spent 30M/HY-24h (M = Cu, Ce, Mn, Fe) catalysts after 24 h of continuous reaction. The red line represents the temperature programming profile.

To elucidate the fundamental causes of deactivation, thermogravimetric analysis (TGA) was performed on the spent catalysts (Figure 9). The weight loss in the critical high-temperature region (300–600 °C), which corresponds to the combustion of hard coke, provides a quantitative measure of catalyst coking. Notably, the mass loss in this region followed the order: 30Cu/HY (2.91 wt%) < 30Fe/HY (3.35 wt%) < 30Mn/HY (4.25 wt%) < 30Ce/HY (4.80 wt%). The 30Cu/HY-24h catalyst exhibited the minimum amount of carbonaceous deposition, confirming that its superior redox property effectively facilitates the deep oxidation of intermediates, thereby inhibiting coke precursor formation. Conversely, the significantly higher weight losses for 30Ce/HY-24h (4.80 wt%) and 30Mn/HY-24h (4.25 wt%) corroborate their more pronounced deactivation, as excessive coke accumulation physically encapsulates active centers.

**Figure 10:**
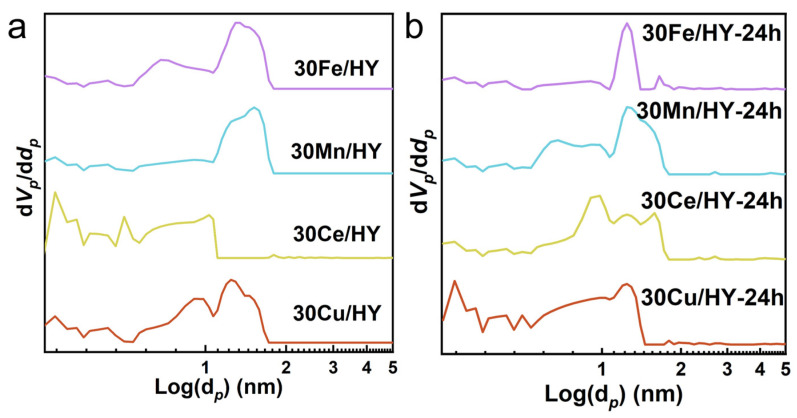
Full-range pore size distribution curves (NLDFT model) of (**a**) fresh 30M/HY catalysts and (**b**) spent 30M/HY-24h catalysts after 24 h of reaction (M = Cu, Ce, Mn, Fe).

The structural impact of this coking is further evidenced by the NLDFT pore size distributions (Figure 10). While the fresh catalysts (Figure 10a) possess a well-defined hierarchical pore structure, the spent catalysts (Figure 10b) show dramatic changes. For 30Ce/HY-24h and 30Mn/HY-24h, which suffered from the heaviest coking (4.80 and 4.25 wt%, respectively), the characteristic micropore peaks (Log(d*_p_*) < 0.5) were almost completely obliterated. This confirms that the accumulated coke occupies the internal zeolite channels, leading to severe pore blockage. In stark contrast, 30Cu/HY-24h, with its minimal carbon deposit (2.91 wt%), successfully preserved its primary microporous architecture. This integrated analysis demonstrates that the high chlorine-resistance and stability of Cu/HY originate from its ability to maintain an open pore network by minimizing carbonaceous residues through efficient deep oxidation. Furthermore, the exceptional preservation of the intrinsic microporous framework in the spent 30Cu/HY catalyst also indirectly confirms its robust structural resistance against potential dealumination caused by the in situ generated HCl.

### 3.7. Exhaust Continuity Monitoring

**Figure 11:**
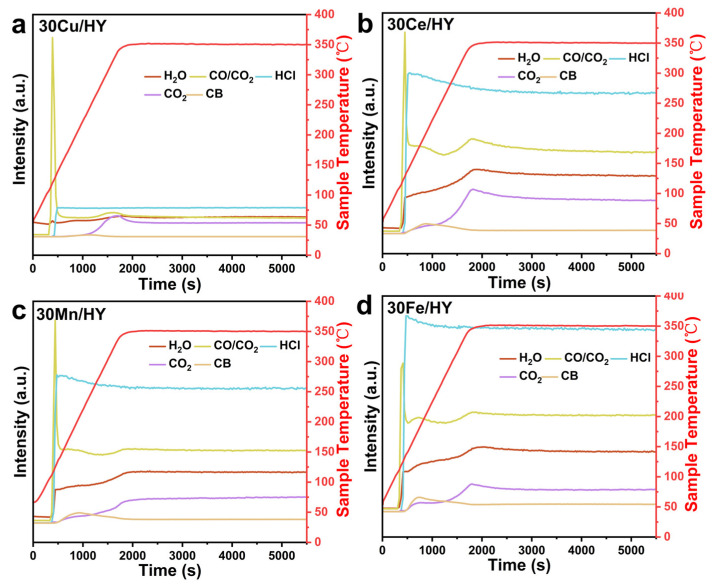
Variation in product distribution during CB catalytic decomposition of 30M/HY catalysts (M = Cu (**a**), Ce (**b**), Mn (**c**), Fe (**d**)) across the temperature range of 50 to 350 °C.

The evolution of key gaseous products (H_2_O, CO, HCl, CO_2_) during the catalytic decomposition of CB over the 30M/HY catalysts (M = Cu, Ce, Mn, Fe) was monitored in real-time using a CATLAB microreactor system coupled with mass spectrometry, across a temperature range of 50 to 350 °C (Figure 11). During the temperature ramp, the signal intensities for all four products exhibited considerable variation. When the temperature stabilized at 350 °C, a steady-state product distribution was achieved. This dynamic behavior suggests a temperature-dependent shift in the dominant reaction mechanism. At lower temperatures, the reaction is primarily mediated by surface-adsorbed reactive oxygen species (e.g., O^−^, O_2_^−^). With increasing temperature, surface lattice oxygen (O^2−^) becomes mobile and contributes directly to the oxidation process. CO and CO_2_ are formed via the incomplete and complete oxidation of the aromatic ring, respectively, reflecting the local availability of active oxygen at the catalyst surface. A characteristic transient spike in the CO (and correspondingly, a lower intensity spike for CO_2_) signal was observed immediately upon CB introduction for all catalysts, followed by rapid decay. This is attributed to a transient local depletion of active oxygen upon initial contact with the high concentration of reactant, which temporarily limits the deep oxidation of CO to CO_2_. H_2_O is generated via the reaction of hydrogen atoms from the decomposing CB molecule with either lattice oxygen or gaseous O_2_. HCl formation follows the cleavage of the C-Cl bond. The Brønsted acid sites on the HY zeolite, along with protons from co-generated water, facilitate the combination of chlorine species with H^+^ to form HCl, thereby promoting its desorption and mitigating catalyst chlorination.

### 3.8. In Situ DRIFTS Spectra and Mechanism Discussion

**Figure 12:**
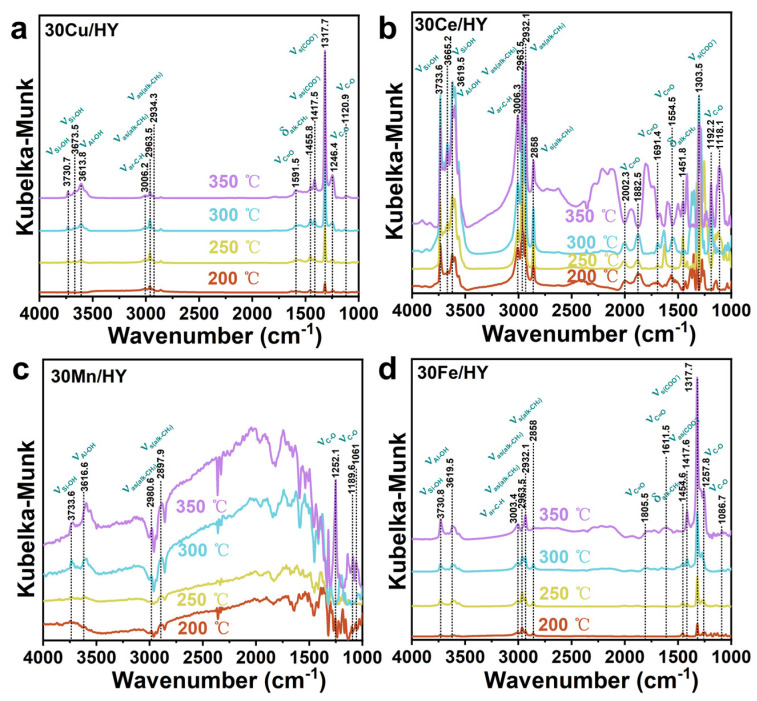
In situ DRIFTS spectra of 30M/HY catalysts (M = Cu (**a**), Ce (**b**), Mn (**c**), Fe (**d**)) for CB over a temperature range of 200 to 350 °C.

Figure 12 presents the in situ DRIFTS spectra acquired on the surfaces of the 30M/HY catalysts (M = Cu, Ce, Mn, Fe) during CB decomposition over a temperature range of 200 to 350 °C. A broad feature in the region of 3734–3613 cm^−1^ is assigned to O–H stretching vibrations of surface silanol (Si-OH) and aluminol (Al-OH) groups [25,34]. The evolution of this band with temperature reflects hydrogen bonding with water produced from the reaction, which modulates the apparent concentration of surface hydroxyls. The aromatic C-H stretching vibrations, observed around 3005 cm^−1^ [41,42], serve as a probe for surface aromatic intermediates. For 30Cu/HY, the near-constant intensity of this band with rising temperature indicates a steady-state concentration, suggesting efficient conversion of these species. In stark contrast, the progressive intensity increase observed for 30Ce/HY, 30Mn/HY, and 30Fe/HY points to significant accumulation of aromatic intermediates on these surfaces. The emergence of characteristic aliphatic bands confirms aromatic ring-opening. These include methylene asymmetric/symmetric stretches (2981–2963 cm^−1^, 2898–2858 cm^−1^), methyl asymmetric stretches (2935–2932 cm^−1^), and methylene bending vibrations (1456–1451 cm^−1^) [42]. For 30Cu/HY, 30Ce/HY, and 30Fe/HY, the intensities of the methylene bands first increase (accumulation > consumption) and then diminish at 350 °C, signifying their subsequent deep oxidation. In contrast, for 30Mn/HY, these bands intensify continuously with temperature, suggesting a slower consumption rate relative to accumulation. A similar trend is observed for the methyl group bands: stable intensity on 30Cu/HY versus a progressive increase on the other catalysts, highlighting the more efficient processing of aliphatic fragments on 30Cu/HY. A broad, complex absorption spanning 2572–2073 cm^−1^, characteristic of cumulated C≡C and conjugated C=C stretching vibrations [41,42], is prominent for 30Ce/HY, 30Mn/HY, and 30Fe/HY but negligible for 30Cu/HY. This indicates significant formation of unsaturated carbonaceous deposits on the former catalysts. Bands in the 2003–1805 cm^−1^ region correspond to C=O stretches of aldehyde/ketone carbonyls [41,42]. A notable red shift of this band for 30Ce/HY suggests a perturbation of the carbonyl group’s electronic environment, likely due to strong interaction with the ceria surface or extended conjugation [41]. Vibrations in the 1692–1591 cm^−1^ range are characteristic of quinoid C=O groups [42], confirming the formation of benzoquinone-type intermediates via partial ring oxidation. These carbonyl-containing intermediates are detected on all catalyst surfaces. Further oxidation leads to the formation of carboxylate species (COO^−^), evidenced by asymmetric and symmetric stretching vibrations at ~1417.5 cm^−1^ and 1318–1303 cm^−1^, respectively [41,42]. An additional band at ~1554 cm^−1^, assigned to the carbonyl vibration of metal-coordinated carboxylates [41], confirms the binding of these intermediates to the respective metal centers (Cu, Ce, Mn, Fe). Finally, the appearance of C-O stretching vibrations in the regions of 1258–1246 cm^−1^ (phenol/alcohol), 1193–1189 cm^−1^ (ether/alcohol), and 1121–1061 cm^−1^ (primary alcohol) [41] signifies the formation of various oxygenates (phenols, alcohols, ethers) as products of progressive ring oxidation and cleavage.

**Figure 13:**
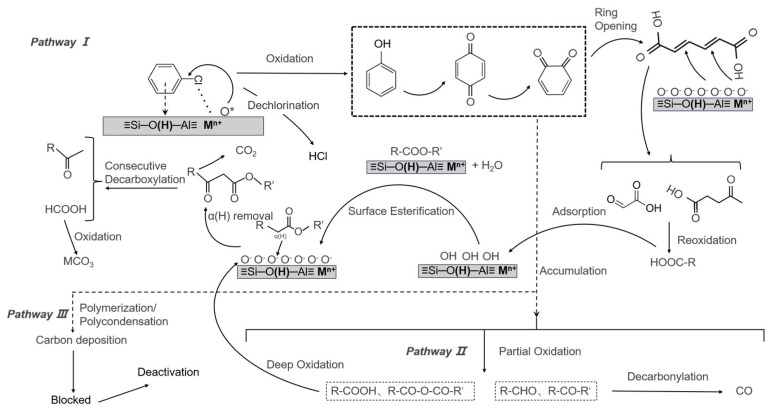
Schematic illustration of the reaction mechanism for the catalytic decomposition of 30M/HY catalysts (M = Cu, Ce, Mn, Fe).

Integrated analysis of online mass spectrometry and in situ DRIFTS reveals that the primary gaseous products are H_2_O, CO, HCl, and CO_2_. Surface-bound intermediates from incomplete oxidation include phenols, aldehydes, carboxylic acids, esters, and ketones, while carbonates are identified as terminal surface species from complete oxidation. Drawing upon these observations and supporting literature, we propose the predominant reaction pathways and elucidate the deactivation mechanisms for CB decomposition over the 30M/HY catalysts. The proposed dominant reaction pathway (Pathway I in Figure 13) initiates with the adsorption and activation of CB. The aromatic ring interacts with Brønsted acid sites (≡Si–OH–Al≡) of the HY zeolite, while the chlorine atom coordinates to Lewis acid sites (metal cations, M^n+^). This dual interaction significantly polarizes and weakens the C-Cl bond, rendering the aromatic carbon electrophilic. Rather than undergoing complete heterolytic cleavage to form an unstable phenyl cation, the activated aromatic ring is concurrently attacked by adjacent reactive surface oxygen species in a concerted nucleophilic-like substitution. This process facilitates the cleavage of the C-Cl bond and directly yields a surface phenolate intermediate (which acts as the precursor to phenol [41]), while the dissociated chloride ion combines with a proton from a neighboring Brønsted acid site to form and desorb as HCl. Subsequent oxidation converts phenol to p-benzoquinone, isomerizing to o-benzoquinone. Ring-opening of o-benzoquinone produces muconic acid, whose C=C bonds are cleaved by oxidative attack to yield short-chain dicarboxylic acids (e.g., glyoxylic acid) [42]. Further oxidation of aldehydes generates carboxylic acids (R–COOH). These acids can esterify with surface hydroxyls, and the resulting esters undergo oxidative decarboxylation (via β-ketoester intermediates) to release CO_2_ and shorten the carbon chain. This cycle continues until formate is formed and ultimately oxidized to surface carbonate. Parallel side pathways (Pathways II and III, Figure 13) contribute to product distribution and deactivation. In Pathway II, phenolic/quinoid intermediates from Pathway I can be over-oxidized to yield a mixture of carboxylic acids, anhydrides, aldehydes, and ketones. While some acids re-enter the main oxidation cycle, aldehydes and ketones may undergo decarbonylation to release CO, which can subsequently be oxidized to CO_2_ if sufficient active oxygen is available. Concurrently, Pathway III involves the polymerization and condensation of phenolic/quinone species, leading to the formation of polyaromatic coke precursors. Catalyst deactivation is primarily attributed to the coverage of active sites by residual surface species. These include partially oxidized intermediates and stable carbonate deposits originating from incomplete combustion, as well as the polymeric carbonaceous species formed via Pathway III, which collectively block access to the active metal centers and acid sites.

## 4. Conclusions

A series of xM/HY catalysts (x = 5, 10, 20, 30, 40; M = Cu, Ce, Mn, Fe) were synthesized via wet impregnation. Initial activity screening identified Cu/HY and Ce/HY as the most promising catalysts for CB decomposition. Among them, the optimized 30Cu/HY catalyst demonstrated the most exceptional performance, achieving near 100% CB conversion at 300 °C alongside outstanding 24 h continuous stability. Comprehensive characterization revealed that the superior performance of Cu/HY fundamentally originates from a powerful dual-site synergy. Quantitative Py-IR analysis confirmed extensive solid-state ion exchange, generating robust Lewis acid centers (Cu-Y structures) that synergize with zeolitic Brønsted acid sites to efficiently polarize and cleave C-Cl bonds. Moreover, XPS and H_2_-TPR analyses indicated that 30Cu/HY possesses an exceptionally high relative concentration of surface-active oxygen (O_β_*/O*_all_ = 0.54), ensuring a continuous and abundant supply of highly reactive surface oxygen which underpins its exceptional low-temperature deep-oxidation capability. Based on an integrated analysis of online mass spectrometry, in situ DRIFTS, and relevant literature, we propose a detailed reaction network for CB decomposition over these catalysts. Key surface-bound intermediates detected include phenols, aldehydes, carboxylic acids, esters, and ketones, with carbonates identified as terminal oxidation products. While catalyst deactivation in other metal oxides is primarily attributed to the blocking of active sites by the accumulation of partially oxidized intermediates and stable carbonaceous deposits (originating from incomplete combustion), TGA and NLDFT analyses clearly demonstrate that the strong deep-oxidation capacity of Cu/HY continuously mineralizes these carbonaceous precursors. This efficiently minimizes coke deposition and preserves the hierarchical pore architecture, ultimately ensuring its outstanding long-term durability.

## Figures and Tables

**Table 1:** Textural properties (S_BET_), actual metal loadings (ICP-OES), and relative atomic ratios of surface oxygen species (XPS) for the HY and 30M/HY catalysts.

Catalyst	S_BET_ (m^2^/g)	*C_a_* (wt%)	*C_t_* (wt%)	O_α_/O_all_	O_β_/O_all_	O_γ_/O_all_
HY	686.47	/	/	/	/	/
30Cu/HY	416.06	19.18	21.81	0.13	0.54	0.33
30Ce/HY	408.24	21.35	21.92	0.21	0.31	0.48
30Mn/HY	387.26	18.03	20.97	0.16	0.28	0.56
30Fe/HY	385.69	20.93	20.99	0.24	0.33	0.43

**Table 2:** Quantitative analysis of Brønsted (B) and Lewis (L) acid site concentrations and B/L ratios for HY and 30M/HY catalysts at varying desorption temperatures.

Catalyst	Temperature (°C)	L-Acid (μmol/g)	B-Acid (μmol/g)	B/L
HY	25	720.3	269.85	0.37
	300	55.59	199.36	3.58
	350	45.23	178.34	3.94
30Cu/HY	25	191.70	24.72	0.12
	300	143.98	18.79	0.13
	350	93.35	8.57	0.09
30Ce/HY	25	400.92	199.36	0.49
	300	122.10	143.13	1.17
	350	30.19	110.09	3.64
30Mn/HY	25	113.91	13.66	0.11
	300	25.51	12.23	0.47
	350	7.55	10.37	1.37
30Fe/HY	25	406.16	90.65	0.22
	300	117.43	75.98	0.64
	350	43.15	67.14	1.55

## Data Availability

The original contributions presented in this study are included in the article/Supplementary Material. Further inquiries can be directed to the corresponding authors.

## Supplementary Materials

The following supporting information can be downloaded at: https://doi.org/10.5281/zenodo.19203955 (accessed on 23 April 2026), Figure S1: SEM-EDS mapping of 30M/HY catalysts (M = Cu, Ce, Mn, Fe).; Figure S2: N_2_ adsorption/desorption isotherms of HY (a) and 30M/HY catalysts (M = Cu (b), Ce (c), Mn (d), Fe (e)); Figure S3: Cu LMM spectra of 30Cu/HY.
